# Effect of a Mental Stimulation Program of Computer and Internet Learning on Cognitive Functions and Wellbeing in Older Community-Dwelling Mexicans

**DOI:** 10.3390/brainsci9070151

**Published:** 2019-06-27

**Authors:** José Miguel Sánchez-Nieto, María de la Luz Martínez-Maldonado, María Montero-López Lena, Víctor Manuel Mendoza-Núñez

**Affiliations:** 1 Doctoral Program in Psychology, National Autonomous University of Mexico, Mexico 04510, Mexico; 2 Research Unit on Gerontology, FES Zaragoza, National Autonomou, University of Mexico, Mexico 04510, Mexico; 3 Faculty of Psychology, National Autonomous University of Mexico (UNAM), Mexico 04510, Mexico

**Keywords:** mental stimulation, computer and internet, learning, cognitive functions, older community-dwelling Mexicans

## Abstract

Background: It has been reported that Mental Stimulation (MS) has a positive effect on cognitive functions and wellbeing. In this sense, different training activities have been proposed for MS such as theater, learning a new language, playing a musical instrument and computing, however, there are few studies on older adults in Latin American countries. For this reason, the purpose of the present study was to determine the effect of a mental stimulation program (MSP) of computer and Internet learning on cognitive functions and wellbeing in older community-dwelling Mexicans. Method: A quasi-experimental pilot study was carried out in a convenience sample of 27 adults aged 60 to 69 years, without knowledge of the use of computers and Internet, without chronic non-communicable diseases, depression or cognitive impairment. Two groups were formed: (i) experimental (EG), *n* = 16 and (ii) control (CG), *n* = 11. The EG participated in an MSP in which 20 theoretical/practical sessions of two hours each were given, two times a week, on computer and Internet. The CG did not participate in any scheduled activity. All participants were measured before and after the intervention program in processing speed (PS), cognitive inhibition (CI), working and episodicmemory (WM and EM), visuospatial processing (VP), life satisfaction (LS) and positive and negative emotions (PE and NE). Results: After participation in the MSP, the EG showed significantly higher scores on the EM and VP tests compared to the CG (*p* < 0.05). **Conclusions**: Our findings suggest that an MSP of computer and Internet learning improves episodicmemory and visuospatial processing in older community-dwelling Mexicans.

## 1. Introduction

Mental stimulation (MS) refers to interventions that promote participation in intellectually stimulating activities to maintain or improve the cognitive functions of older adults. In this sense, it has been shown that different training activities of MS such as theater, playing a musical instrument, learning a new language or competence such as computation and Internet, can improve or maintain cognitive functions [1]. It has also been proposed that MS could improve wellbeing [2,3].

For MS to have an effect on cognitive functions, we must consider that the task has to encourage the use of cognitive processes, self-initiated processing, new learning and adjust the difficulty of the task for the person to perform a high cognitive effort, without exceeding its limits [3,4]. Also, the increase in wellbeing can be facilitated by the incorporation of positive comments focused on the task, and an environment that promotes autonomy, competence and relationship [5].

In the present study, computer and Internet learning was chosen as an activity for the mental stimulation program (MSP), considering that a high percentage of the Mexican elderly population does not have the basic knowledge about the use of computers and the Internet. Hence, this activity fulfills the requirement of being a new knowledge, it also requires self-initiated processing and may have an increasing level of difficulty, as well as having been associated whith greater wellbeing [6]. It has also been shown that the use of the computer is positively related to performance in cognitive tests and a lower probability of developing mild cognitive impairment [7,8].

Knowledge about the effect of computing on the cognitive functions of older adults is not entirely consistent. Slegers et al. (2009) found that the use of computation does not have an effect on cognitive functions and wellbeing [9]. However, Klusmannet al. (2010) found that learning the use of the computer and the Internet has a positive effect on long-term memory [10]. Also, Chan et al. (2016) observed that teaching the use of tablets, speed of processing and episodic memory of the participants improve, but not working memory and visuospatial processing [11]. In adittion, Vaportzis et al. (2016) conducted a similar study, finding a positive effect on processing speed, but not in working memory [12]. Therefore, considering the findings of the quoted studies, we infer that MSP through computer and Internet learning will maintain or improve cognitive functions and wellbeing in older adults.

In this context, the purpose of the present study is to determine the effect of an MSP of computer and Internet learning on cognitive functions and wellbeing in older community-dwelling Mexicans. 

## 2. Method

### 2.1. Design and Participants

A quasi-experimental pilot study was carried out in a convenience sample of 27 adults over 60 to 69 years of age in Mexico City without chronic uncontrolled non-communicable diseases such as type 2 diabetes mellitus, arterial hypertension, Parkinson’s disease, psychiatric disorders, epilepsy (or stroke), depression (score ≥5 on the abbreviated Scale of Depression Geriatric Yesavage) [13] or cognitive impairment (≤20 in the Montreal Cognitive Assessment) [14]. All participants signed the informed consent. The project was approved by the Ethics Committee of the Universidad NacionalAutónoma de México (UNAM), Zaragoza Campus (Project PAPIME PE305516).

**Figure 1:**
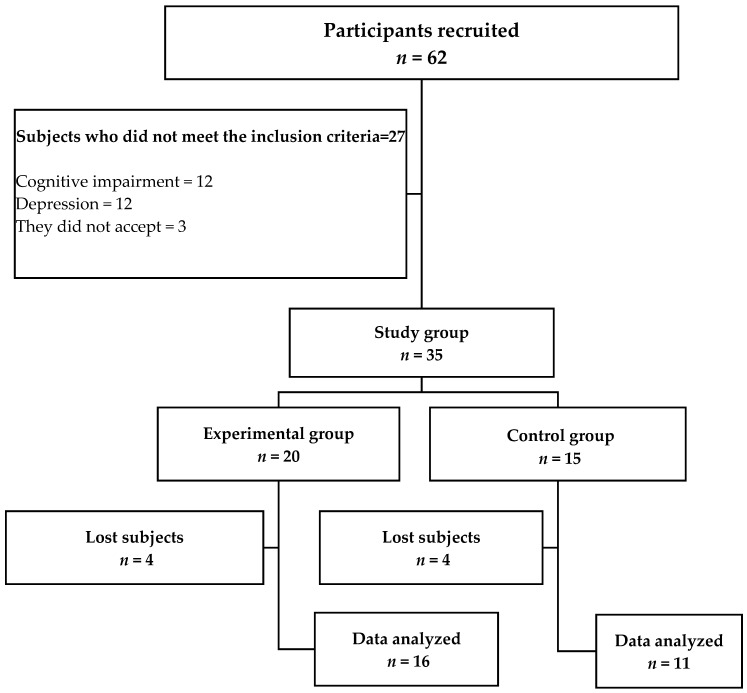
Outline of the study.

An open invitation was held in Mexico City and 62 people responded, of which 35 met the inclusion criteria and agreed to participate in the investigation. In this sense, 20 subjects accepted to participate in the MSP 20 program (EG, experimental group), of which 4 did not conclude with the program, and 15 subjects were included in the control group (CG) because they did not have time to participate in the MSP, although they agreed to be evaluated at the beginning and after ten weeks, only 11 subjects participated in the second evaluation (Figure 1). For this reason, the data analysis was carried out in the following groups: (i) experimental group (EG) *n* = 16: 2 men and 14 women, age 64.3 ± 3.4 years, schooling 8.9 ± 2.6 years; (ii) control group (CG) *n* = 11: 3 men and 8 women, age 64.3 ± 3.2, schooling 9.1 ± 3.3 years.

### 2.2. Intervention

The MSP consisted of a 40-hour workshop, distributed in 20 sessions, two times a week (ten weeks). The workshop was taught in groups of 10 participants. The instructor wasthe principal investigator. The themes are shown in Table 1, although they were individually adapted according to the interests of the participants.

To encourage the increase of cognitive functions in the implementation of the MSP, the following strategies were incorporated: i) the use of the computer constantly; ii) teach something new in each session, either in procedures or information; iii) that will explore how to perform the activities (self-initiated processing) and iv) adjust the difficulty of the task at a high level. Based on the theory of self-determination [5], to improve the subjective wellbeing during the program i) the value of the task was recognized by itself and extrinsic motivation was avoided, for example giving rewards or punishmentsii) positive comments were expressed focusing on the skills of the participants, showing emphasis on the process of doing the activity rather than on the finality, as well as avoiding making destructive criticisms or devalorizing comments to the participants; iii) they were encouraged to choose goals, preferably based on small tasks, feedback was given suggesting elements that would allow them to identify their skills and limits, given the necessary time to answer questions and carry out activities, help was promoted between the participants of the group and suggestions were given to find the information that interested them.

### 2.3. Instruments

All participants were measured before and after the intervention program in processing speed (PS), cognitive inhibition (CI), working and episodicmemory (WM and WE), visuospatial processing (VP), life satisfaction (LS) and positive and negative emotions (PE and NE). The evaluation was carried out by two psychologists, who received a training of 10 hours, in which theoretical and technical aspects of the application and qualification of the instruments were discussed.In addition, each evaluator made two supervised applications to different people of the research participants. The application was made two weeks before and two weeks after the intervention. It has been made individually in two sessions of one hour. The instruments were applied face to face with printed documents. 

For the evaluation of cognitive processes, the following instruments were used:

- Processing speed: Test Stroop Color [15]; Symbol Digit Modalities Test (SDMT) [16]; Trail Making Test A [17].
- Working memory: Paced Auditory Serial Addition Test (PASAT) three sec [17]; Trail Making Test B [17]; Letter–Number Sequencing of the WAIS-IV (LNS) [16].
- Cognitive inhibition: Stroop color-word test [15].
- Episodic memory: trial 5, 7 and learnining (sum of trial one to five) of the Rey auditory verbal learning test [18].
- Visuospatial processing: Matrix reasoning of the WAIS-IV [16].

Wellbeing was assessed using the life satisfaction scale (α = 0.83) [19] and the Positive and Negative Affect Schedule, positive emotions (α = 0.85–0.90) and negative emotions (α = 0.81–0.85) [20].

### 2.4. Data Analysis

For the analysis of the data, the SPSS program, version 20, was used. The means and percentages are reported according to the variables evaluated. The pretest score was subtracted from the posttest of each participant, the means and standard deviations of that difference were obtained for each group. We used repeated measures ANOVA to evaluate changes in outcome variables throughout the intervention period considering statistical significance when *p* < 0.05; effect size was estimated with the eta cuadradoparcial(η_p_^2^).

## 3. Results

### 3.1. Processing Speed

The EG showed an increase in processing speed in comparison with the CG. However, when performing the ANOVA of repeated measures no statistically significant differences were found between the groups in the scores of the Symbol Digit Modalities Test *F*(1,25) = 2.1, *p* = 0.15, η_p_^2^ = 0.08, Trail Making Test A*F*(1,25) = 3.3, *p* = 0.07, η_p_^2^ = 0.12; and Stroop Color *F*(1,25) = 0.22, *p* = 0.63, η_p_^2^ = 0.009 (Table 2).

### 3.2. Working Memory and Cognitive Inhibition

There was an increase in the score in the tests of working memory and cognitive inhibition in the EG, nevertheless, when performing the ANOVA test of repeated measures no statistically significant differences were observed between the groups in the scores of the Trail Making Test B *F*(1,25) = 1.5, *p* = 0.22, η_p_^2^ = 0.05; Letter-Number Sequencing *F*(1,25) = 1.2, *p* = 0.28, η_p_^2^ = 0.04; Paced Auditory Serial Addition Test *F*(1,25) = 1.4, *p* = 0.23, η_p_^2^ = 0.05; and Stroop color-Word*F*(1,25) = 0.1, *p* = 0.74, η_p_^2^ = 0.004 (Table 2). 

### 3.3. Episodic Memory

A statistically significant increase in the test score was observed to measure the episodic memory in the EG compared to the CG. With the ANOVA analysis of repeated measurements was observed effect of the MSP the trial 5 *F*(1,25) = 6.6, *p* = 0.01, η_p_^2^ = 0.21; and learning *F*(1,25) = 11.8, *p* = 0.002, η_p_^2^ = 0.32. (Table 3). 

### 3.4. Visuospatial Processing

A positive effect on visuospatial processing was observed in the EG group compared to the CG group. When performing the repeated measures ANOVA test, a statistically significant increase was found in the evaluation of the Matrix Reasoning test *F*(1,25) = 5.2, *p* = 0.03, η_p_^2^ = 0.17 (Table 3).

### 3.5. Subjective Wellbeing

No statistically significant differences were observed between the EG and CG in the scores of negative emotion test, *F*(1,25) = 0.75, *p* = 0.72, η_p_^2^ = 0.004; positive emotion test,*F*(1,25) = 0.26, *p* = 0.61, η_p_^2^ = 0.01; and satisfaction with life test, *F*(1,25)= 3.7, *p* = 0.06, η_p_^2^ = 0.12 (Table 4).

## 4. Discussion

Human aging is a gradual and adaptive process, characterized by a relative decrease in the biological and cognitive reserve and response to demands to maintain or restore homeostasis, due to morphological, physiological, biochemical, psychological, and social changes, prompted by genetic load and accumulated wear, and the challenges that a person faces throughout their life in a given environment [21]. In this sense, cognitive aging is a complex process in which there is an increase in the accumulation of knowledge and, in turn, a decrease in the capacity to process new information. In addition, processes related to attention such as processing speed, working memory and cognitive inhibition begin to descend from the age of 30, with a lower variability between individuals, instead, cognitive processes related to memory and reasoning begin to descend to 60 years, with greater variability between individuals [22,23].

Knowledge about strategies that can avoid or defer the decline in cognitive functions with aging, mainly those related to care is not conclusive [24]. However, it has been shown that mental stimulation could be a practical and accessible strategy to maintain cognitive functions and that the person continues with their autonomy and independence throughout the life cycle up to old age [25].

In the present study we tried a program of mental stimulation of learning of the use of Internet and the computer, in which we found a positive effect on the episodic memory and the visuospatial processing. The foregoing contrasts with the findings ofSlegers et al. (2009), that the use of the computer does not have an impact on cognitive functions [9]. These differences may be due to the fact that we included specific strategies to maintain the cognitive functions that have been implemented in other mental stimulation interventions [11,12,26,27]. This suggests that to achieve an impact on cognitive functions, not only must a task be performed that is considered intellectually stimulating, but the task has to encourage the use of cognitive processes, self-initiated processing, learning and an adjustment of tasks for a high cognitive effort.

Our results can be explained by considering the scaffolding theory of aging and cognition [28] and adult cognitive plasticity theory [4]. The scaffolding is the recruitment of additional neural networks that support the declining structures whose functioning became noisy, inefficient or both. This is formed by learning something new and activities related to executive functions. The area of the brain in which it develops is mainly the frontal lobe [23]. They can also be explained by the plasticity of the brain, which allows the development of anatomical changes. Plasticity is generated by keeping the demands of the environment high for the individual, in such a way that it can acquire more processing resources or knowledge to adapt to the environment, generating new connections in the brain [4].

We found a positive change in episodic memory and visuospatial processing in the EG; however, no statistically significant differences were observed in the variables related to attention such as working memory, processing speed and cognitive inhibition between the groups (EG and CG). These findings are consistent with what has been reported in other studies, which showed that learning to use a tablet or digital photograph had a positive effect on episodic memory, butnot in attention [11,26].

On the other hand, it has been shown that performing different stimulating activities in supervised programs such as learning computer skills, practicing theater and helping children with their homework has a positive effect on cognitive functions [1]. This supports the proposal that mental stimulation can improve or maintain cognitive functions, considering thedesign of programs with activities that respond to the interest of the population and ensure engagement, adherence and a growing effort [21].

It has been proposed that mental stimulation can improve subjective wellbeing [26]. We design the intervention based on the theory of self-determination to promote wellbeing through positive comments and a structure of activity that promotes autonomy, competence and relatedness. 

After the intervention, we found that the participants of the mental stimulation group did not show statistically significant changes in the scores of Processing Speed, Working Memory and Inhibition tests, and the subjective wellbeing tests. In this sense, it is important to note that the control group self-selected, because they did not agree to participate, because they did not have time. Therefore, their daily activities could have influenced the results, especially if the activities were stimulating, complex and satisfactory, these could influence the cognitive functions and subjective wellbeing. In this regard, it has been pointed out that the types and characteristics of activities that promote subjective wellbeing and cognitive health should be identified, since the resources generated will motivate people to continue with these activities and, in turn, these resources will allow healthier behaviors [29].

Finally, it is important to point out that the present study has some limitations, such as a small sample size, the lack of random assignment of the groups, and the lack of monitoring of the activities that could influence cognitive functions and subjective wellbeing in the control group. In this regard, we suggest continuing with this line of research considering a representative sample size, random assignment of the groups and include in the design the registration and measurement of the activities carried out by the participants in the control group that could influencethe cognitive functions and subjective wellbeing. 

## 5. Conclusions

Our findings suggest that mental stimulation based on the learning of computer and Internet use, have a positive effect episodic memory and visuospatial processing. Thus, this study supports the proposal to recommend the mental stimulation as an alternative to maintain or improve the cognitive functions of older adults. 

## Figures and Tables

**Table 1:** Topics of the computer course.

1. Basic conceptsaboutcomputing	11. Searchfor places onthe Internet
2. Operatingsystem and mouse	12. Visits to virtual museums
3. Keyboard use	13. Searchforinformationpagesonthe Internet
4. Folders and subfolders	14. Internet procedures
5. Folders and subfolders 2	15. Downloadmusicfromthe internet
6. Basic concepts and Internet search	16. Search of activitiesfortheweekend
7. Searchonthe Internet: images	17. Searchforentertainment 1
8. Characteristics of Internet pages	18. Searchforentertainment 2
9. Internet search: videos	19. Searchforentertainment 3
10. Internet search: Games	20. Searchforentertainment 4

**Table 2:** Mean Score of Processing Speed, Working Memory and Inhibition Tests by Study.

	Experimental Group (EG) (*n* = 16)		Control Group (CG) (*n* = 11)		Change EG	Change EC
	Pre	Post	Pre	Post		
Processingspeed						
SDMT	22 ± 6	25 ± 7	20 ± 4	21 ± 5	3.3 ± 3.4	0.4 ± 2.3
TMTA	245 ± 19	256 ± 14	239 ± 19	236 ± 25	11.1 ± 13.1	−3.3 ± 13.7
SC	58 ± 6	60 ± 7	62 ± 6	58 ± 1	2.5 ± 5.3	−4.1 ± 5
Workingmemory						
TMTB	161 ± 51	181 ± 45	151 ± 41	147 ± 47	24.0 ± 6	19.2 ± 5.8
LNS	13 ± 2	14 ± .2	13 ± 3	12 ± 3	1.4 ± 1.7	−1.1 ± 1.7
PASAT 3s	30.7 ± 7	33.4 ± 10.4	29.1 ± 7.4	27.0 ± 9.4	2.7 ± 6.1	−2.1 ± 4.9
Cognitive inhibition						
SC-W	29 ± 6	32 ± 7	30 ± 6	30 ± 6	3.2 ± 3.3	−0.6 ± 5.0

Repeated measures ANOVA *p* < 0.05, PS: Processing speed; WM: Working memory; SDMT: Symbol Digit Modalities Test; TMT A: Trail Making Test A; SC: Stroop color; TMT B: Trail Making Test B; LNS: Letter–Number Sequencing; PASAT: Paced Auditory Serial Addition Test; SC-W:Stroop color-Word

**Table 3:** Mean Score of Episodic Memory and Visuospatial Processing Tests by Study Group.

	Experimental Group (EG) (*n* = 16)		Control Group (CG) (*n* = 11)		Change EG	Change CG
	Pre	Post	Pre	Post		
Episodicmemory						
E5	11 ± 2	13 ± 1	11 ± 2	10 ± 2	2.3 ± 1.8*	−0.3 ± 1.6
Learning	41 ± 6	53 ± 6	40 ± 5	41 ± 7	12.2 ± 7.2*	0.73 ± 5.5
E7	10 ± 2	11.0 ± 1.7	9.2 ± 1.6	10 ± 2	1.2 ± 2.3	0.4 ± 2.3
Visuospatial processing						
MR	9 ± 4	12 ± 4	8 ± 2	8 ± 3	2.9 ± .7*	1.1 ± 0.03

*, Repeated measures ANOVA *p* < 0.05, E5: trial 5; E7: trial 7; MR: Matrix reasoning.

**Table 4:** Mean Score of Negative and Positive Emotion and Satisfaction with Life Tests by Study Group.

	Experimental Group (EG) (*n* = 16)		Control Group (CG) (*n* = 11)		Change EG	ChangeCG
	Pre	Post	Pre	Post		
NE	22.19 ± 8.2	16.7 ± 5.6	22.82 ± 5.6	17.5 ± 6.9	-5.5 ± 6.7	−5.2 ± 4.7
PE	37.1 ± 7.0	41.4 ± 6.5	39.09 ± 5.7	37.1 ± 5.5	4.2 ± 5.2	−1.9 ± 4.8
SL	19.63 ± 2.3	21.4 ± 1.7	18.5 ± 1.9	19.7 ± 2.5	1.7 ± 2.2	1.2 ± 2.2

Repeated measures ANOVA *p* > 0.05, NE: Negative emotion; PE, Positive emotion; SL; Satisfaction with Life.
